# Equitable health services for the young? A decomposition of income-related inequalities in young adults’ utilization of health care in Northern Sweden

**DOI:** 10.1186/s12939-017-0520-3

**Published:** 2017-01-18

**Authors:** Paola A. Mosquera, Anna-Karin Waenerlund, Isabel Goicolea, Per E. Gustafsson

**Affiliations:** Department of Public Health and Clinical Medicine, Unit of Epidemiology and Global Health, Umeå University, Umeå, SE-901 87 Sweden

**Keywords:** Health Inequality, Health care utilization, Horizontal inequity index, Decomposition analysis, Young adults, Sweden

## Abstract

**Background:**

Despite the goal of the Swedish health system to offer health care according to the principle of horizontal equity, little is known about the equality in access to health care use among young people. To explore this issue, the present study aimed i) to assess horizontal inequity in health care utilization among young people in Northern Sweden; and ii) to explore the contribution of different factors to explain the observed inequalities.

**Methods:**

Participants (*N* = 3016 youths aged 16–25 years) came from the “Health on Equal terms” survey conducted in 2014 in the four northernmost counties in Sweden. Concentration indices (C) and horizontal inequity indices (HI) were calculated to measure inequalities in the utilization of two health care services (general practitioners (GP) and youth clinics). The HI was calculated based on health care utilization and variables representing socioeconomic status (household income), health care needs factors and non-need factors affecting health care use. A decomposition analysis was carried out to explain the income-related inequalities.

**Results:**

Results showed a significant positive income-related inequality for youth clinic utilization in women (*C* = 0.166) and total sample (*C* = 0.097), indicating that services were concentrated among the better-off. In contrast, general practitioner visits showed inequality pointing toward a higher utilization among less affluent individuals; significant in women (*C* = −0.079), men (*C* = −0.101) and pooled sample (*C* = −0.097). After taking health care needs into consideration, the utilization of youth clinics remained significantly pro-rich in women (HI = 0.121) and total sample (HI = 0.099); and consistently pro-poor for the GP visits in the pooled sample (HI = −0.058). The decomposition analyses suggest that socioeconomic inequalities explain a considerable portion of the pro-rich utilization of youth clinics services among young women. The corresponding analyses for GP visits showed that need factors and socioeconomic conditions accounted for the pro-poor concentration of GP visits.

**Conclusion:**

The distribution of GP visits among young people in Northern Sweden slightly favored the low-income group, and thus seems to meet the premises of horizontal equity. In contrast, the findings suggest substantial pro-rich horizontal inequity in the utilization of youth clinics among young women, which are largely rooted in socioeconomic inequalities.

## Background

The Swedish health system has as a goal to provide health care according to the principle of horizontal equity [1]; that is, that health care should be provided on equal terms to all individuals, giving priority to those with greatest need of care [2, 3]. However, Sweden seems to fall short of this goal, as suggested by demonstrations of social and income inequalities in health care utilization [1, 4]. These inequalities in health care are also reflected in corresponding inequalities in health [5], which have been observed already among young people [6]. Whereas Sweden has a variety of health services where youths can seek health information and care–*youth clinics* being the best known example–there is no research on whether the health care services for youths live up to the goal of an equitable care. To shed light on this topic, the present study seeks to estimate income-related inequalities in health care utilization among young people in Northern Sweden; and to explain these inequalities through health-related, social and socioeconomic conditions.

Access to primary health care services for youth is an important measure to ensure good health in young people [7] and thereby also to establish a good and ideally equitable health at an early age. However, in many countries young adults report worse access to health care than do older adults [8], and socioeconomic barriers seem to hamper utilization of health services [9, 10]. In Sweden, health care is partly decentralized and the responsibility for provision and coordination lies on the 21 county councils. Young people can seek primary care through primary health care centers (e.g. general practitioners, GPs), which provides primary care to the entire population; and also through youth clinics, which are solely devoted to young people (from adolescence up to approximately age 25 years) [11]. Health care visits to the GP’s are free of charge up to about 20 years of age, and are thereafter subject to subsidized patient charges [12]. Services offered at health care centers cover general health needs, i.e. curative as well as health promotion and preventive services. Youth clinics are always free of charge [11] and their services focus on reproductive and sexual health issues, as well as psychosocial problems such as substance abuse. At certain youth clinics, other aspects of health of young people are also covered, e.g. mental health problems and social relationships. Youth clinics started in 1970 with the intent to serve young peoples’ health need [13], and today they represent a large and unique network with over 250 clinics spread across the country. As such, the youth clinics play an important role in primary prevention during the salient life course period of transition into adulthood.

Swedish studies on inequalities in health care utilization have shown that people with low income indeed visit GPs to a higher degree [14]. However, studies also indicate that this high utilization is mainly explained by the greater health care needs among the poor, and when such differential needs are taken into account, utilization instead favors the well-off [15, 16]. This pattern of overutilization of outpatient care among the rich relative to the poor differs from findings in other Scandinavian countries [17, 18] and most OECD countries [16, 19], where no horizontal inequities have been found after taking health care needs into account. Nevertheless, Swedish and international research has focused on adults [14, 15] or elderly [4]. Little is therefore known about equality in health care utilization among young people in general, and particularly when it comes to the dedicated health services for this group, such as the Swedish youth clinics. As such, it remains unknown whether the goal of the Swedish counties to offer equal and needs-based access to care [2] is met for young people. This knowledge gap seems unfortunate, especially bearing in mind that the well-developed network of youth clinics has a potential for contributing to an equal health for all, during a key stage in life [20].

To contribute to knowledge about horizontal equity in young people’s health care utilization, the objectives of the present study were i) to assess income-related inequalities and horizontal inequity in the utilization of youth clinics and visits to general practitioners among young people (aged 16–25) in Northern Sweden; and ii) to explore the contribution of health-related, social and socioeconomic factors to the observed inequalities in health care utilization.

## Methods

### Study population and data

This study used data from the 2014 Health on Equal Terms survey (HET–“Hälsa på lika villkor” in Swedish), conducted in the four northernmost counties of Sweden: Västernorrland, Jämtland/Härjedalen, Västerbotten, and Norrbotten. The HET survey is carried out by the Swedish National Institute of Public Health in partnership with the county councils/regions and Statistics Sweden. Sampling was done in two steps. First, as part of the national HET survey, a national random sample covering the entire Sweden was drawn, of which those residing in any of the four northern Swedish counties were eligible for inclusion in the present study (*N* = 1789 invited). Second, the four counties opted to make a regionally expanded random sample (*N* = 50,300 invited), stratified into 276 strata by county, municipality, gender and age, and using the same questionnaire as the national survey. The overall response rate was approximately 50%. The total study population comprised 25,667 individuals aged 16–84 years from the total population of Northern Sweden, of whom 96% of the respondents were sampled specifically for the regional survey in the four counties, and 4% were sampled for the national survey. For the present study, we selected individuals aged 16–25 years, resulting in a sample size of 1742 females and 1274 males, in total 3016 persons.

Data for the survey were collected through a postal questionnaire covering different health domains such as physical and mental health, use of pharmaceuticals, contact with health care services, dental services, health behaviors, financial conditions, work and occupation, work environment, safety, health status and social relationships. In addition, national register variables from the total population registers of Statistics Sweden, such as income, country of birth, and educational level, were linked to the survey data through the unique Swedish Personal Identity Number.

### Variable definition

The variables used in this study were classified into four categories: a) *health care utilization* (outcomes), b) *socioeconomic status* (ranking variable), c) *health care needs factors*, and d) *non-need factors* affecting health care use.

*Health care utilization* was measured by *Youth clinic utilization* and *GP visits*, derived from the questions: a) “Have you visited a youth clinic in the last 3 months?” and b) “Have you visited a GP at a health care center in the last 3 months?”, respectively. Both variables were coded as yes = 1 and no = 0.

*The socioeconomic status* variable used to rank the population was household disposable income. This measure represents the amount of money available to a household for spending on goods or services after income taxes and all positive and negative transfers (such as debts) have been accounted for. For the decomposition analysis, the variable was divided into quintiles.

*Need factors* are biological determinants such as sex, age and health status which are used as proxies of “health care need” [21]. In this study we included self-rated health and reported diseases/symptoms as health status variables for both youth clinics and GP visits. As the focus of the youth clinics have gradually broadened to cover other aspects of health of young people, we included alcohol problems, drug use and violence as additional variables to capture “health care need” for the utilization of this service.

*Sex* was defined as male/female. *Age* was categorized into three groups: 16–18/19–22/23–25 years. *Self-rated health* was categorized into four groups: very good/good/fair and poor/very poor. *Long-term illness* was measured by the question “Do you have any long term illness, discomfort following an accident, any reduced physical function or any other long term health problem?” (Yes/No). *Mental health* was measured by the General Health Questionnaire (GHQ)-12 [22, 23], which comprises twelve items each coded on a four-level Likert scale, which are summed up into an index (range = 36; Cronbach α = 0.89). *Health complaints* were based on ten self-reported general symptoms (covering musculoskeletal pain in neck; back; and extremities; headache; worries; tiredness; sleeping difficulties; eczema; tinnitus; bowel symptoms), scored on three-level Likert scales and summed up into one index (range = 20; Cronbach α = 0.72). *Alcohol problems* were defined by the binary response to the question “Would you like to reduce your alcohol consumption?” (Yes/No). *Drug use* was defined by a positive response to either of the two following questions: “Have you in the last 12 months used hash or marihuana? and “Have you in the last 12 months used any narcotics other than hashish or marijuana?” (Yes/No). *Violence* was defined by a binary response to either of the following two questions: “Have you during the last 12 months been subjected to physical violence?” and “Have you during the last 12 months been subjected to a threat or menace of violence so that you were scared?” (Yes/No).

*Non-need factors* considered in this analysis were socioeconomic predictors for health care utilization, as well as income. *Educational level* was categorized into low educational level (compulsory school or shorter) and medium/high (upper secondary school and higher education). *Place of birth* was divided in two categories: being born in Sweden/other country. *Type of occupation* was classified into four categories: studying/working/being unemployed or in a labor market program and other (sickness benefits/disability pension, long-term sick leave, taking care of the home). *Cash margin* was measured by the question “would you manage to find 15000SEK in 1 week in the case of an unforeseen situation?” (Yes/No). *Availability of youth clinic* in the municipality was defined as Yes/No. For the analysis of GP visits, a variable representing *rurality and availability of hospitals* at municipal level was also created, comprising four categories: municipalities with population >50,000 with hospitals, 10–50,000 with hospitals, 10–50,000 without hospitals, <10,000 without hospitals.

### Statistical analysis

To address the first aim, the concentration index (C) and horizontal inequity index (HI) were calculated to estimate inequalities in health care utilization, without (C) and with (HI) differential need of health care taken into account. To address the second aim, a decomposition analysis was carried out to quantify the contribution of need and non-need factors to the observed income-related inequality. To avoid overestimation of need/non-need factors contribution owing to correlation with income, income was also included as one of the decomposition factors [24].

In this study, the C represents an unadjusted (crude) measure of inequality in health care utilization, while the HI is the need-adjusted version of the unadjusted concentration index. The C of health care utilization was calculated by the following equation [21]:

1 $$ C=\frac{2}{\mu }\ cov\ \left(h,r\right) $$

Where *h* is the health care variable; *μ* is the mean or proportion of the health care variable; and *r* is the rank of individuals according to their socioeconomic status (household income), from the most disadvantaged to the least disadvantaged. The value of the C can vary between −1 and +1, where a negative (positive) value indicates that the outcome of interest is concentrated among individuals with relatively low (high) income, and C equals zero under perfect equality. As the health outcome was binary, we applied the normalization proposed by Wagstaff et al. to the concentration index [21, 25].

To quantify the contribution of need and non-need factors to the observed inequality in health care utilization, we conducted a decomposition analysis [21]. The decomposition of the C is based on regression analysis of the relationship between a health variable and a set of *k* determinants. According to the World Bank technical notes, decomposition of a non-linear outcome requires some linear approximation that restores the underlying assumptions of the decomposition method [21]. As suggested by Doorslaer et al. [19, 21], we used the linear approximation of a probit model with the marginal/partial effects evaluated at means, which is expressed by the formula:

2 $$ {Y}_i={\propto}^m+{\displaystyle {\sum}_j{\beta}_j^m{X}_{ij}} + {\displaystyle {\sum}_k{\gamma}_k^m{Z}_{ij}} + {\varepsilon}_i $$

The concentration index for *Y*_*i*_ *, C*, can thus be written as:

3 $$ C={\displaystyle {\sum}_j\left({\beta}_j^m{\overline{x}}_j/\mu \right)}{C}_j+{\displaystyle {\sum}_k\left({\gamma}_k^m{\overline{Z}}_k/\mu \right)}{C}_k+G{C}_{\varepsilon }/\mu $$

Where *μ* is the mean of *Y*_*i*_ (health care utilization variable); *C*_*j*_ and *C*_*k*_ are the concentration index of *X*_*j*_ (need factors) and *Z*_*k*_ (non-need factors); *β*_*j*_^*m*^ and *γ*_*k*_^*m*^ are the marginal effects, *dy*/*dx*_*j*_ and *dy*/*dz*_*k*_, of each need (*x*) and non-need (*z*) factor; ${\overline{X}}_j$ and ${\overline{Z}}_k$ are the mean of *X*_*j*_ and Z_k_ (need and non-need factors); the products $\left({\beta}_j^m{\overline{x}}_j/\mu \right){C}_j$ and $\left({\gamma}_k^m{\overline{Z}}_k/\mu \right){C}_k$ are the contributions of a need factor (*j)* and a non-need factor (*k)* to the unadjusted concentration index, respectively; and *GC*_*ε*_ is the generalized concentration index of the error term. Both the absolute contribution (i.e. expressed on the same unit as the concentration index) and relative contribution (percentages of the total concentration index) to the unadjusted inequality in the health care utilization are presented in the result section. A positive (negative) contribution indicates that the variable operates towards pro-rich (pro-poor) distribution of health care visits.

To measure the inequity in health care utilization, we calculated the HI by subtracting the absolute contributions made by need factors in Eq. (3) from the unadjusted concentration index [19, 21]. The HI thus captures the socioeconomic inequity in health care utilization while controlling for the effects of health care needs. Equivalent to the interpretation of the concentration index, a positive (negative) value of HI indicates horizontal inequity favoring the better-off (worse-off), and a zero index value indicates that health care utilization and needs are proportionally distributed across the income distribution [19]; that is, that health care is utilized according to needs. We obtained *P*-values for the HI using the indirect standardization method for measuring horizontal inequity, as suggested by Doorslaer and Wagstaff [19, 21, 26]. All analyses were performed on women and men separately to capture potential gender-specific patterns.

## Results

The characteristics of the study population are presented in Table 1. Overall, GPs were visited more frequently (25.5%) than were youth clinics (14.8%) over the last 3 months. Women used health care services considerably more frequently than did men, which was particularly marked for youth clinics (23% in women vs 4% in men) and less so for GP visits (31% vs 18%). The living conditions were fairly similar between women and men. However, women tended to report worse health than men across all different health measures.

**Table 1:** Description of characteristics of study population by gender

	Women		Men		Total	
	*N*	%	*N*	%	*N*	%
Health care utilization						
Youth clinic visits	381	22.72	50	4.07	431	14.8
GP visit	520	30.79	222	18.09	742	25.5
Age						
16–18 years	413	23.7	344	27.0	757	25.1
19–22 years	800	45.9	569	44.7	1369	45.4
23–25 years	529	30.4	361	28.3	890	29.5
Education level						
Low level	750	49.9	528	51.2	1278	50.5
Medium/High level	752	50.1	503	48.8	1255	49.6
Place of birth						
Sweden	1639	94.1	1167	91.6	2806	93.0
Other country	103	5.9	107	8.4	210	7.0
Household income (SEK) ^a^						
Lowest quintile	111,933	48345.0	126,208	73681.6	115,034	56312.0
2	252,912	48765.8	316,961	47173.2	280,603	49413.0
3	432,905	48357.7	481,338	39808.3	454,306	45566.6
4	577,964	38541.3	602,779	37261.8	588,938	36752.0
Highest quintile	847,184	346586.2	864,326	319545.7	855,054	334927.7
Cash margin						
Yes	1069	61.97	855	67.75	1924	64.4
No	656	38.03	407	32.25	1063	35.6
Type of occupation						
Working	461	26.46	352	27.63	813	27.0
Studying	933	53.56	650	51.02	1583	52.5
Unemployed	162	9.3	163	12.79	325	10.8
Other	186	10.68	109	8.56	295	9.8
Municipality size of residence						
> 50,000 habitants with hospital	443	25.43	334	26.22	777	25.8
10,000–50,000 habitants with hospital	323	18.54	224	17.58	547	18.1
10,000–50,000 habitants without hospital	290	16.65	228	17.9	518	17.2
< 10,000 habitants without hospital	686	39.38	488	38.3	1174	38.9
Youth clinic						
Yes	1131	65.11	826	64.94	1957	65.0
No	606	34.89	446	35.06	1052	35.0
Self-rated health						
Very good	420	24.32	426	33.68	846	28.3
Good	933	54.02	655	51.78	1588	53.1
Fair	314	18.18	154	12.17	468	15.6
Poor/Very poor	60	3.48	30	2.37	90	3.0
Long term illness	524	30.39	345	27.29	869	29.1
Mental health^a^	22.64	5.8	20.65	4.6	21.80	5.4
Health complaints^a^	13.92	3.1	12.32	2.5	13.24	3.0
Alcohol problems	141	10.4	120	12.8	261	11.4
Drug use	69	4.0	72	5.7	141	4.7
Violence	141	8.1	113	8.9	254	8.4

^a^ mean (standard deviation)

**Fig. 1:**
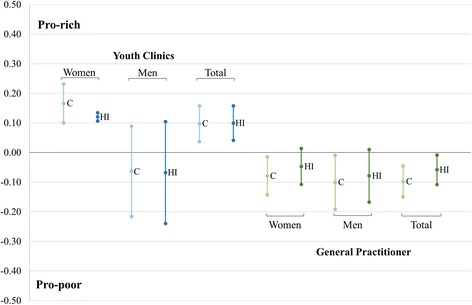
Concentration indices (C) and Horizontal inequity indices (HI) for health care utilization among young people

Corresponding to the first aim, the unadjusted concentration indices (C) and needs-adjusted horizontal inequity indices (HI) for youth clinics utilization and GP visits are shown in Fig. 1. A significant pro-rich distribution for youth clinics utilization was observed in women (*C* = 0.166) and the total sample (*C* = 0.097), thus demonstrating that services were concentrated among individuals belonging to higher income households. A nonsignificant tendency in the other direction was observed in men (*C* = −0.063). In contrast, general practitioner visits showed inequality in an opposite direction, pointing toward a higher utilization among individuals belonging to lower income households; significant in women (*C* = −0.079), men (*C* = −0.101) and in the pooled sample (*C* = −0.097). After taking the health care needs into account, the utilization of youth clinics remained significantly pro-rich in women (HI = 0.121) and total sample (HI = 0.099); and consistently pro-poor for the GP visits, which was significant only in the pooled sample (HI = −0.058) but pointed in the same direction for both women (HI = −0.047) and men (HI = −0.079).

**Fig. 2:**
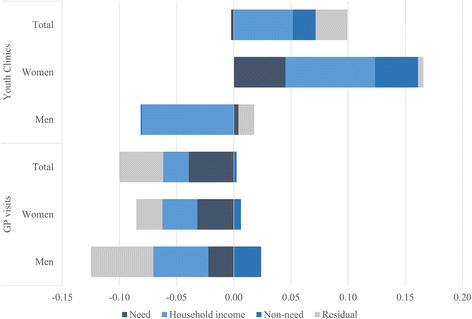
Summary of decomposition of income related inequalities in health care utilization among young people

Corresponding to the second aim, results of the decomposition analysis are summarized in Fig. 2, and reported in detail in Table 2 (youth clinics) and Table 3 (GP visits) (note that the decomposition of the nonsignificant inequality in youth clinic utilization in men should be interpreted with caution, but the values are reported for the sake of completeness). The analyses of youth clinics indicated that need, non-need and household income to a large degree accounted for the pro-rich concentration in women, who were the greatest users of this service (Table 1). In contrast, in the pooled sample the contributions of need factors were slightly offsetting the contributions of the non-need and household income (Fig. 2). Moreover, the need variables included in the analysis explained a relatively small proportion of the inequality favoring the well-off (27% in women), whereas income and non-need factors played a greater explanatory role (70 and 73% in the women and pooled sample, respectively). Among the need factors, older age was the major contributor to the observed pro-rich distribution, and to a lesser extent long-term illness, whereas the other factors displayed little explanatory value. Among the non-need factors, low household income made the largest contribution to the pro-rich inequality, with additional contributions from education, occupation and country of birth. The contribution of having a youth clinic in the municipality of residence, on the other hand, did not contribute independently to the inequality (Table 2).

**Table 2:** Decomposition of the concentration index of youth clinic utilization among young people in Northern Sweden

	Women					Men					Total				
	Coeff	Elast	CI	Cont to C	%	Coeff	Elast	CI	Cont to C	%	Coeff	Elast	CI	Cont to C	%
Sex															
Men															
Women											0.213***	2.264	−0.013	−0.031	−31.4
Age															
16–18 years															
19–22 years	−0.072	−0.145	0.104	−0.015	−9.0	−0.022	−0.242	0.016	−0.004	6.1	−0.051*	−0.155	0.066	−0.010	−10.6
23–25 years	−0.139**	−0.186	−0.306	0.057	34.3	−0.053*	−0.366	−0.079	0.029	−45.8	−0.098***	−0.195	−0.215	0.042	43.3
Self-rated health															
Very good															
Good	0.040	0.095	0.012	0.001	0.7	−0.002	−0.024	−0.020	0.000	−0.7	0.020	0.072	−0.002	0.000	−0.2
Fair	0.027	0.022	−0.074	−0.002	−1.0	−0.014	−0.043	−0.129	0.006	−8.7	0.002	0.002	−0.098	0.000	−0.2
Poor/Very poor	−0.037	−0.006	−0.081	0.000	0.3	−0.013	−0.007	−0.143	0.001	−1.6	−0.023	−0.005	−0.104	0.000	0.5
Long term illness	−0.082**	−0.471	−0.014	0.006	3.8	−0.004	−0.119	−0.008	0.001	−1.4	−0.043**	−0.376	−0.011	0.004	4.3
Mental health	0.002	0.231	−0.004	−0.001	−0.6	0.002	0.789	−0.012	−0.010	15.2	0.002	0.305	−0.009	−0.003	−2.9
Health complaints	0.002	0.132	−0.016	−0.002	−1.3	0.001	0.228	−0.012	−0.003	4.2	0.001	0.064	−0.017	−0.001	−1.1
Alcohol problems	0.072	0.033	0.029	0.001	0.6	0.011	0.033	−0.117	−0.004	6.2	0.043	0.033	−0.037	−0.001	−1.3
Drug use	0.106	0.018	0.068	0.001	0.8	0.048*	0.066	−0.084	−0.006	8.7	0.093**	0.029	−0.005	0.000	−0.2
Violence	0.057	0.020	−0.115	−0.002	−1.4	0.039	0.086	−0.085	−0.007	11.6	0.047	0.027	−0.098	−0.003	−2.7
Subtotal Need				0.045	27.2				0.004	−6.5				−0.002	−2.3
Household income															
Lowest quintile	−0.109**	−0.096	−0.795	0.076	46.1	0.007	0.037	−0.790	−0.029	45.6	−0.049**	−0.065	−0.794	0.052	53.3
2	−0.070**	−0.061	−0.397	0.024	14.7	0.030	0.150	−0.394	−0.059	93.0	−0.031	−0.024	−0.483	0.012	11.9
3	−0.070	−0.061	0.000	0.000	0.0	0.016	0.078	0.001	0.000	−0.1	0.031	0.030	−0.228	−0.007	−7.1
4	−0.064	−0.056	0.397	−0.022	−13.5	0.004	0.019	0.396	0.008	−11.9	−0.035	−0.045	0.106	−0.005	−4.9
Highest quintile															
Education level															
Low level															
Medium/High level	−0.056	−0.370	−0.053	0.020	11.9	0.008	0.281	−0.003	−0.001	1.4	−0.023	−0.236	−0.033	0.008	8.0
Type of occupation															
Studying															
Working	−0.094**	−0.109	−0.127	0.014	8.4	−0.007	−0.049	0.048	−0.002	3.7	−0.049**	−0.090	−0.050	0.004	4.6
Unemployed	0.011	0.005	−0.101	0.000	−0.3	−0.007	−0.021	−0.151	0.003	−4.9	0.008	0.006	−0.116	−0.001	−0.7
Other	0.007	0.003	−0.018	0.000	0.0	−0.002	−0.004	−0.116	0.000	−0.7	0.005	0.003	−0.057	0.000	−0.2
Place of birth															
Sweden															
Other country	−0.154	−0.719	−0.007	0.005	3.2	−0.005	−0.144	−0.020	0.003	−4.7	−0.079	−0.567	−0.013	0.008	7.8
Cash margin															
Yes															
No	−0.025	−0.042	−0.053	0.002	1.3	0.005	0.042	−0.101	−0.004	6.7	−0.009	−0.023	−0.076	0.002	1.8
Youth clinic															
Yes															
No	−0.044	−0.067	0.043	−0.003	−1.7	0.005	0.041	−0.002	0.000	0.2	−0.017	−0.040	0.022	−0.001	−0.9
Subtotal Non-Need				0.116	70.0				−0.081	128.3				0.072	73.6
Total				0.161					−0.077					0.069	
Residual				0.005					0.014					0.028	
C				0.166***					−0.063					0.097**	
HI				0.121***					−0.068					0.099**	

*Coeff* Marginal effects from the probit model, *Elast* elasticity, *CI* Concentration index of the social determinants, *Cont to C* Contribution to the overall concentration index, *%* unadjusted percentage calculated on the overall explained portion of the C

* 0.01 ≤ *p* < 0.05; ** 0.001 ≤ *p* < 0.01; *** *p* < 0.001

**Table 3:** Decomposition of the concentration index of GP visits among young people in Northern Sweden

	Women					Men					Total				
	Coeff	Elast	CI	Cont to C	%	Coeff	Elast	CI	Cont to C	%	Coeff	Elast	CI	Cont to C	%
Sex															
Men															
Women											0.105***	0.650	−0.013	−0.009	9.0
Age															
16–18 years															
19–22 years	0.026	0.039	0.104	0.004	−5.1	−0.029	−0.073	0.016	−0.001	1.1	0.000	0.000	0.066	0.000	0.0
23–25 years	0.010	0.010	−0.306	−0.003	4.0	−0.037	−0.058	−0.079	0.005	−4.6	−0.014	−0.016	−0.215	0.003	−3.5
Self-rated health															
Very good															
Good	0.122***	0.214	0.012	0.003	−3.3	−0.021	−0.061	−0.020	0.001	−1.2	0.055*	0.115	−0.002	0.000	0.3
Fair	0.226***	0.133	−0.074	−0.010	12.5	−0.010	−0.007	−0.129	0.001	−0.9	0.120**	0.074	−0.098	−0.007	7.4
Poor/Very poor	0.372***	0.042	−0.081	−0.003	4.3	0.074	0.010	−0.143	−0.001	1.4	0.249**	0.029	−0.104	−0.003	3.2
Long term illness	0.134***	0.566	−0.014	−0.008	9.7	0.130***	0.913	−0.008	−0.007	6.9	0.135***	0.687	−0.011	−0.008	7.9
Mental health	−0.005*	−0.405	−0.004	0.002	−2.2	0.007**	0.844	−0.012	−0.010	10.2	−0.001	−0.112	−0.009	0.001	−1.1
Health complaints	0.023***	1.026	−0.016	−0.016	20.5	0.011*	0.774	−0.012	−0.009	9.0	0.019***	1.014	−0.017	−0.017	17.2
Subtotal Need				−0.032	40.4				−0.022	22.0				−0.039	40.4
Household income															
Lowest quintile	0.066	0.043	−0.795	−0.034	43.5	0.054	0.060	−0.790	−0.048	47.3	0.046	0.036	−0.794	−0.028	29.2
2	−0.040	−0.026	−0.397	0.010	−13.0	0.036	0.040	−0.394	−0.016	15.6	−0.042	−0.019	−0.483	0.009	−9.5
3	−0.006	−0.004	0.000	0.000	0.0	−0.063	−0.069	0.001	0.000	0.1	0.004	0.002	−0.228	0.000	0.5
4	−0.025	−0.016	0.397	−0.006	8.1	0.035	0.039	0.396	0.015	−15.3	−0.031	−0.024	0.106	−0.003	2.6
Highest quintile															
Education level															
Low level															
Medium/High level	−0.052	−0.252	−0.053	0.013	−17.0	0.063*	0.521	−0.003	−0.002	1.6	−0.001	−0.003	−0.033	0.000	−0.1
Type of occupation															
Studying															
Working	0.047	0.040	−0.127	−0.005	6.5	0.001	0.002	0.048	0.000	−0.1	0.033	0.035	−0.050	−0.002	1.8
Unemployed	−0.017	−0.005	−0.101	0.001	−0.7	0.006	0.004	−0.151	−0.001	0.6	−0.002	−0.001	−0.116	0.000	−0.1
Other	0.019	0.007	−0.018	0.000	0.2	−0.068	−0.032	−0.116	0.004	−3.7	−0.010	−0.004	−0.057	0.000	−0.2
Place of birth															
Sweden															
Other country	−0.092	−0.315	−0.007	0.002	−3.0	−0.142*	−0.852	−0.020	0.017	−17.3	−0.100*	−0.419	−0.013	0.006	−5.7
Cash margin															
Yes															
No	0.006	0.008	−0.053	0.000	0.5	−0.012	−0.022	−0.101	0.002	−2.2	−0.003	−0.004	−0.076	0.000	−0.3
Municipality size of residence															
> 50,000 habitants with hospital															
10,000–50,000 habitants with hospital	0.018	0.011	0.017	0.000	−0.2	0.071	0.069	0.067	0.005	−4.6	0.035	0.025	0.037	0.001	−0.9
10,000–50,000 habitants without hospital	0.046	0.025	−0.089	−0.002	2.8	0.110**	0.108	−0.008	−0.001	0.9	0.077**	0.052	−0.051	−0.003	2.7
< 10,000 habitants without hospital	−0.060	−0.077	0.029	−0.002	2.8	0.027	0.058	−0.017	−0.001	1.0	−0.024	−0.036	0.008	0.000	0.3
Subtotal Non-Need				−0.024	30.5				−0.024	24.0				−0.020	20.3
Total				−0.056					−0.046					−0.059	
Residual				−0.023					−0.054					−0.038	
C				−0.079**					−0.101*					−0.097***	
HI				−0.047					−0.079					−0.058**	

*Coeff* Marginal effects from the probit model, *Elast* elasticity, *CI* Concentration index of the social determinants, *Cont to C* Contribution to the overall concentration index, *%* unadjusted percentage calculated on the overall explained portion of the C

* 0.01 ≤ *p* < 0.05; ** 0.001 ≤ *p* < 0.01; *** *p* < 0.001

The corresponding analyses for GP visits showed that need and household income largely accounted for the pro-poor concentration of GP visits, while the non-need factors instead offset the inequality (Fig. 2). In contrast to the youth clinics, inequalities in GP visits in women and in the pooled sample were more explained by need factors (40% in both women and pooled sample) than by non-need factors (31% in women; 20% in the pooled sample), and by an equal share in men (22% need; 24% non-need). The pro-poor inequalities were explained by greater health care needs among those with health complaints, worse self-rated health and long-term illnesses. In men, poor mental health was also among the most important contributors. From the non-need factors, the pro-poor health care inequality was mostly explained by household income inequalities; in women and total sample also slightly by occupation and municipality of residency, whereas education (women) and place of birth (men) instead were offsetting the inequality (Table 3).

## Discussion

To our knowledge, this is the first study estimating and decomposing inequalities in health care service utilization among young people. The results suggest that whereas ordinary primary care is fairly equitable and adhere to the principle of horizontal equity among young adults in Northern Sweden, the use of youth clinics among women, who stand for the largest share of youth clinic utilization, is substantially skewed towards the financially well-off. Furthermore, the decomposition analyses suggest that socioeconomic inequalities explain a considerable portion of this relative overutilization of youth clinics services among the more affluent young women.

The substantial pro-rich inequalities in the youth clinic utilization among young women are both perplexing and concerning. This is particularly true considering the contrasting pro-poor inequalities seen for GP visits, and the fact that there indeed is a greater health care need among poor young people, including for sexual and reproductive health services [11, 27]. The observed pattern for GP visits is in accordance with previous studies analyzing inequalities in health care utilization in the general population in Sweden, in other Scandinavian countries like Norway [17] and Denmark [18], as well as in most of the OECD countries [16, 19]. After adjusting for need factors, the inequality still favored the low-income group, suggesting that the likelihood of young people visiting a GP appears to be distributed chiefly according to need, and not by affluence. This finding is in general concordance with other Scandinavian studies reporting no significant horizontal inequity in the GP visits [17, 18], but diverge with the positive horizontal inequity found for doctor visits (GPs and specialists) among Swedish adults, reported by Doorslaer et al. in 2006 [16] and Agerholm et al. [15]. Our study adds to this meagre literature by suggesting that GP use is fairly equitable among young adults in Northern Sweden.

The fact that the GP visits meet the premises of horizontal equity in young people could be an expected effect of having a health system with universal coverage, where individual health care visits are greatly subsidized and free of charge until the age of 20. In theory, a free-of-charge service should not create differences in utilization between socio-economic groups. Nevertheless, the larger pro-rich inequality in the utilization of youth clinics, a service that is free of charge regardless of age, defies this rationale. Apparently, social inequalities in health care do not necessarily abide such rational assumptions, and equal access across social groups does not seem to necessarily result in equitable utilization. Even if the services do not involve patient fees, other financial barriers could remain, such as costs for transportation or prescribed medicines, which are not easy to afford for the less affluent.

Shedding light on possible explanations to the pro-rich inequality in youth clinic utilization, the decomposition analysis pointed out household income, education, occupation and to a lesser degree place of birth as the most important contributors. Such economic, educational and ethnic barriers have indeed been described in other countries as hindering young adults [9, 10] and the general population [16, 28] to use health care services, although others have found that socioeconomic barriers are minor when it comes to young people’s health care access [29]. Another possible explanation for the contrasting inequalities could be the different types of services offered by health care centers and youth clinic. For example, youths with greater health care needs and with more severe illness, which are more frequent among the poor, would more likely seek the curative services offered by a GP instead than visit a youth clinic, where the services are more focused on health promotion and prevention. In other words, it is possible that it is easier to reach healthier youths who also are more likely to be well-off with the type of services offered by the youth clinics. This is concerning since health promotion that is unintentionally selective for the already healthy and well-off could create “intervention generated inequalities” [30], thus perpetuating instead of reducing health inequalities.

The Swedish Society for Youth Centers (FSUM) has already pointed out that utilization is not equal for all youths, mainly focusing on the poorer utilization of young men [31], a gendered pattern also evident in the present study. However, income inequalities in utilization have not received much interest. In Sweden, most studies on health care inequalities concern the general population, or are designed in manners precluding examinations of inequalities in utilization [32]. This means that neither general health care use among young people nor the special services provided by the youth clinics are well monitored when it comes to their potential inequitable utilization. As stated by the World Health Organization [33, 34], initiatives developed to improve young people’s health–of which the Swedish youth clinics are an example–need to consider not only health issues but also the potential importance of socioeconomic determinants. Our findings exemplify the necessity of such considerations, even in a health system and societal context which would be assumed to be able to provide equitable care, and concerning a health service without direct economic barriers.

Income inequalities may also hinder youth-friendly service utilization, an issue that requires more exploration, particularly considering that most of the available evidence has instead focused on service-based issues, e.g. cultural aspects and providers’ attitudes [35, 36]. Given the more equal utilization of GPs than of youth clinics, one could speculate whether the services currently offered by the youth services would be more equally utilized if instead provided by the primary health care centers. However, there is no evidence that this would be the result, and if the root of the problem instead is the nature of the services offered to youth, it is likely that no improvement in equity would be gained of such a major re-organization. In the end, there is no evidence in support of any specific solution. Further studies are therefore required to identify possible barriers to the use of the services among the underserved population of youth–men and less affluent women. Such a direction would help researchers and policy makers to find ways to promote equitable utilization of youth clinics between different socio-economic groups. Likewise, an equity lens [37] should be implemented into the youth clinic services, including careful monitoring and active strategies to reduce the observed socioeconomic inequalities in utilization, focusing on identifying mechanisms that either promote, or fail to prevent, the utilization of health services among the less advantaged youth.

### Methodological considerations

The strengths of the present study include a population-based sample of young people, the use of survey data in combination with national register data, and the application of rigorous statistical methods. However, the study was cross-sectional, which naturally precludes any inferences concerning causality, and the fairly low response rate of 50% may introduce selection bias. Although it is unlikely that a slight underrepresentation of e.g. economically disadvantaged people would seriously impact on the concentration of health care use across the income spectrum, the extent to which any selection bias is reflected in estimates and inferences is ultimately unknown. Furthermore, the social and public health landscape, as well as the prerequisites for equity in health care utilization from the sparsely populated northern Sweden are expected to differ from the ones in the more populated south; thus, generalization of the findings should be done with a certain amount of caution.

When it comes to measures, the income measure captured household rather than individual income, since many young people are expected to still be financially dependent on their parents. However, it should be noted that precision of household income as a measure of the socioeconomic situation of the youths could potentially vary by age. For example, the youngest youths still living at home with parents are more likely to belong to a high-income household, while older youths who are more likely to live independently would more likely belong to low-income households. As the questionnaire did not include information on the complete set of family members, we were unable to calculate equalized income, which would have been a more appropriate measure of socioeconomic status.

Another methodological challenge concerns formulating need variables for young people in general, and for capturing needs for the youth clinics in particular. Although the common set of self-assessed health, age and sex have usually been sufficient to describe health care need in the overall population [16], the small proportion explained by the need variables in the present study suggests that other measures are required to make the need adjustment in young people. The questionnaire did for example not cover information related to sexual and reproductive health, which is likely to be one main reason for seeking youth clinic care [11]. Nevertheless, since poor sexual and reproductive health is expected to be overrepresented among the poor rather than among the rich, it is unlikely that a more inclusive coverage of need factors would have impacted on the pro-rich inequity estimates for youth clinics. It is however possible that a better needs measurement would have reduced the pro-poor inequalities in GP visits even further.

## Conclusion

Visits to general practitioner among young people in Northern Sweden was slightly more frequent among poorer youth, and thus seems to meet the premises of horizontal equity. In contrast, we found substantial pro-rich horizontal inequity in women’s utilization of youth clinics, which were largely rooted in socioeconomic inequalities. These results are surprising considering that the service is free of charge, and that the need for health care is greater among the poor. Our study strongly implies the need for implementing an equity lens into the youth clinic provision of Sweden, lest they may remain a service for the well-off which in the long run may entrench health inequalities, particularly among young women.

## Abbreviations

C: Concentration index
FSUM: The Swedish Society for Youth Centers
GP: General practitioner
HET: Health on Equal Terms survey
HI: Horizontal inequity index
OECD: Organization for Economic Co-operation and Development
